# A hybrid approach to dynamic cognitive psychometrics: Dynamic cognitive psychometrics

**DOI:** 10.3758/s13428-023-02295-y

**Published:** 2024-01-10

**Authors:** Charlotte C. Tanis, Andrew Heathcote, Mark Zrubka, Dora Matzke

**Affiliations:** 1 Department of Psychology, University of Amsterdam, Postbus 15916, 1001 NK Amsterdam, Netherlands; 2 Department of Psychology, University of Newcastle, Newcastle, Australia

**Keywords:** Cognitive psychometrics, Choice behavior, Response inhibition, Stop-signal paradigm, Reaction times

## Abstract

Dynamic cognitive psychometrics measures mental capacities based on the way behavior unfolds over time. It does so using models of psychological processes whose validity is grounded in research from experimental psychology and the neurosciences. However, these models can sometimes have undesirable measurement properties. We propose a “hybrid” modeling approach that achieves good measurement by blending process-based and descriptive components. We demonstrate the utility of this approach in the stop-signal paradigm, in which participants make a series of speeded choices, but occasionally are required to withhold their response when a “stop signal” occurs. The stop-signal paradigm is widely used to measure response inhibition based on a modeling framework that assumes a race between processes triggered by the choice and the stop stimuli. However, the key index of inhibition, the latency of the stop process (i.e., stop-signal reaction time), is not directly observable, and is poorly estimated when the choice and the stop runners are both modeled by psychologically realistic evidence-accumulation processes. We show that using a descriptive account of the stop process, while retaining a realistic account of the choice process, simultaneously enables good measurement of both stop-signal reaction time and the psychological factors that determine choice behavior. We show that this approach, when combined with hierarchical Bayesian estimation, is effective even in a complex choice task that requires participants to perform only a relatively modest number of test trials.

*Cognitive psychometrics* (Batchelder, 1998, 2009; Kellen, Winiger, Dunn, & Singmann, 2021; Vandekerckhove, 2014) measures mental capacities in a way that is informed by information-processing models from experimental psychology and the neurosciences. Importantly, if these models are to support valid inferences about psychological and neural processes, their parameters must have sufficiently good measurement properties, i.e., it should be possible to obtain accurate and precise parameter estimates from data in realistic research settings. Originally developed for categorical data using multinomial processing tree models (Riefer & Batchelder, 1988), cognitive psychometrics has increasingly taken on a dynamic aspect, taking into account how long it takes for cognitive processes to produce behavior. This has been the case both in reinforcement learning models accounting for dependencies between sequences of discrete choices (O’Doherty, Cockburn, & Pauli, 2016) and in evidence-accumulation models for the response time (RT) associated with each choice (Donkin & Brown, 2018), and more recently in these two frameworks combined (Miletić et al., 2021; Pedersen, Frank, & Biele, 2017). Dynamic cognitive psychometrics has underpinned advances in areas ranging from theory-driven computational psychiatry (Huys, Maia, & Frank, 2016; Weigard, Heathcote, Matzke, & Huang-Pollock, 2019) to aging research (Garton, Reynolds, Hinder, & Heathcote, 2019; Ratcliff, Thapar, & McKoon, 2001). However, as dynamic cognitive psychometrics is increasingly applied to the complex tasks that are of interest in applied domains, maintaining good measurement properties becomes increasingly challenging.

We develop a new approach to address these challenges in a paradigm that is widely used to measure executive (i.e., non-habitual) control with respect to response inhibition, the stop-signal paradigm. In this paradigm, participants make a series of choice responses and at unpredictable intervals are required to withhold their response when a stop signal appears some time after the choice stimulus. As well as being of interest in its own right—with many applications across clinical, cognitive, and life-span psychology and the neurosciences (e.g., Aron & Poldrack, 2006; Badcock, Michie, Johnson, & Combrinck, 2002; Bissett & Logan, 2011; Fillmore, Rush, & Hays, 2002; Forstmann et al., 2012; Hughes, Fulham, Johnston, & Michie, 2012; Matzke, Hughes, Badcock, Michie, & Heathcote, 2017; Schachar, Mota, Logan, Tannock, & Klim, 2000; Schachar & Logan, 1990; Verbruggen, Stevens, & Chambers, 2014; Williams, Ponesse, Schachar, Logan, & Tannock, 1999; Skippen et al., 2020)—this paradigm highlights a key challenge for cognitive psychometrics in general: strong trade-offs among parameters due to the need to simultaneously estimate many latent (i.e., not directly observable) quantities.

In many applications of cognitive modeling in general and cognitive psychometrics in particular, hierarchical Bayesian estimation (Gelman et al., 2014; Heathcote et al., 2019) can help to address this challenge through the extra constraint afforded by shrinkage, that is, constraint afforded by assuming commonalities among participants. However, this solution can become ineffective due to a problem specific to dynamic contexts that use RTs and estimate a lower bound (i.e., “shift”) parameter for the distribution of RTs. Estimating parameter-dependent lower bounds violates one of the regularity conditions that assures the optimality of likelihood-based estimation, that distribution support is known. In particular, a lower bound that has to be estimated from data implies that the support of the distribution (i.e., range over which the distribution has non-zero likelihood) is unknown as it varies for different values of the lower-bound parameter. The lower-bound problem has been extensively studied in the statistical literature with respect to shifted lognormal, Weibull, and gamma distributions. In these cases, a shift estimate at the minimum observation has an infinite likelihood, rendering maximum likelihood estimates of the model’s other parameters inconsistent (Cheng & Amin, 1983). A similar problem can also cause Bayesian (hierarchical) estimation of process models of the stop-signal paradigm (e.g., Logan, Van Zandt, Verbruggen, & Wagenmakers, 2014) to be inconsistent (Matzke, Logan, & Heathcote, 2020).

Here we propose, and demonstrate the efficacy of, a solution to this estimation problem in the stop-signal paradigm. The solution illustrates a general lesson, that dynamic cognitive psychometric models with desirable measurement properties can require modeling solutions where some psychological-process components are replaced by descriptive elements that provide a flexible statistical description of the data but are not committed to any particular psychological process.

In the next section, we detail the stop-signal paradigm and the way in which purely process models requiring estimates of shift parameters fail. We then introduce our “hybrid” modeling solution that mixes descriptive and process-based elements. Next, we investigate whether the hybrid stop-signal model can provide an accurate characterization of empirical data from a complex experimental design with factors that are known to selectively influence only particular subsets of the process component’s parameters. If the hybrid model is valid, parameter estimates should reflect the selective influence of experimental manipulations on the psychological processes they are supposed to represent. Hence, a parameterization subject to these selective influence constraints should accurately fit the data. We then report the results of a parameter-recovery study showing that Bayesian methods require only a modest number of test trials to accurately estimate the model’s parameters in this complex design. We end by discussing how our proposed methodology can be applied more broadly to expand the purview of dynamic cognitive psychometrics.

## The stop-signal paradigm

**Fig. 1:**
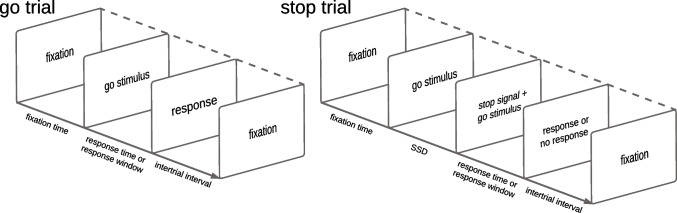
Schematic representation of the trial types in the stop-signal paradigm. SSD: stop-signal delay. Figure available at https://flic.kr/p/2n1oRSe under a CC-BY 2.0 license (https://creativecommons.org/licenses/by/2.0/)

In the stop-signal paradigm (see Fig. 1; for in depth treatments, see Logan, 1994; Matzke, Verbruggen, & Logan, 2018; Verbruggen et al., 2019) participants typically make a series of well-practiced and easy choices (e.g., press left button for left-pointing arrow and right button for right-pointing arrow). On a minority of trials, this primary “go” task is interrupted by a stop signal requiring participants to withhold their response to the choice “go” stimulus. Typically, participants can successfully withhold their response when the delay between the onset of the go stimulus and the stop signal (i.e., stop-signal delay; SSD) is relatively short, and they fail when the stop signal comes close to the execution of the choice response.

Inhibitory ability is quantified by the stop-signal reaction time (SSRT), the time between the appearance of the stop signal and the successful inhibition of the choice response. As SSRT is, by definition, a latent quantity (i.e., it cannot be directly observed because the response is withheld), its measurement requires a psychometric model. With few exceptions, this role is fulfilled by Logan and Cowan’s (1984) “horse-race” model: an independent race between a go process triggered by the go stimulus and a stop process triggered by the stop signal. A response is executed if the go racer wins, and is withheld if the stop racer wins. This simple model enables non-parametric estimation of summary measures of SSRT with minimal but often inaccurate assumptions about the race times. For instance, the “integration” method makes the unrealistic assumption that the finishing time of the stop runner is constant. The most popular version of the “mean” method treats stopping latency as a random variable but incorrectly assumes that the function relating SSD and the probability of stopping, and hence the distribution of RTs on go trials, is symmetrical (for details, see Matzke et al., 2018).

Using central tendency measures to summarize RTdistributions—such as go RT and SSRT distributions—can be misleading (Heathcote, Popiel, & Mewhort 1991; Matzke & Wagenmakers, 2009). The Bayesian parametric “BEESTS” approach (Matzke, Dolan, Logan, Brown, & Wagenmakers, 2013; Matzke et al., 2013) addresses this problem by estimating the full distribution of go RTs and SSRTs assuming that these follow an ex-Gaussian distribution. The ex-Gaussian distribution arises from the convolution of a Gaussian and an exponential random variable, and has been frequently used as a *descriptive* purely statistical account of RT distributions (Heathcote et al., 1991; Hohle, 1965; Matzke & Wagenmakers, 2009; Ratcliff, 1978). The $\mu$ and $\sigma$ parameters quantify the mean and standard deviation of the Gaussian component, and $\tau$ reflects the slow tail of the distribution. Although it provides an excellent descriptive model of RT distributions, the psychological interpretation of the ex-Gaussian parameters is ambiguous, and so is discouraged (Matzke & Wagenmakers, 2009).

BEESTS has been extended to enable the identification of the relative contribution of inhibition failures and attention failures to stop-signal performance using a dynamic multinomial processing tree representation (Matzke, Love, & Heathcote, 2017). It has also been extended to model the inhibition of difficult, and hence error-prone, choices, by characterizing the go process in terms of one runner corresponding to each choice option (Matzke, Curley, Gong, & Heathcote, 2019). The BEESTS approach has excellent psychometric properties and has been successfully applied to stop-signal data in various research areas (e.g., Chevalier, Chatham, & Munakata, 2014; Colzato, Jongkees, Sellaro, van den Wildenberg, & Hommel, 2014; Jana, Hannah, Muralidharan, & Aron, 2020; Matzke et al., 2017; Skippen et al., 2019; Skippen et al., 2020; Weigard et al., 2019).

### Stop-signal process models

The computational power of race or “winner-takes-all” architectures (Maass, 2000; Šíma & Orponen, 2003; Heathcote & Matzke, 2022) makes them widely applicable across psychology and the neurosciences. As opposed to descriptive models, the runners are interpreted as psychological and neural processes that accumulate noisy evidence and finish when the accrued amount reaches a threshold (Donkin & Brown, 2018). RT corresponds to the time for the first runner to finish (decision time) plus the time required for the sensory encoding of the stimulus and the production of a motor response after the winner finishes (non-decision time). Non-decision time (or its lower bound if it is assumed to be variable) constitutes the potentially problematic shift parameter that must be estimated from data due to systematic differences between individuals and groups. For example, it is well documented that older participants have longer non-decision times than younger participants (e.g., Garton et al., 2019; Ratcliff et al., 2001).

A variety of racing evidence-accumulation models have been proposed, typically assigning one racer to each response option and differing in assumptions about evidence, such as whether it varies from moment-to-moment (diffusive noise), from trial-to-trial, or both, in the degree of correlation of evidence among accumulators, and whether it accrues linearly or non-linearly. One of the more commonly applied models for binary choice, the Wiener diffusion model, assumes linear accumulation of perfectly negatively correlated diffusive noise, which is formally equivalent to a single process diffusing between two thresholds (Stone, 1960). Other widely used models assume independent races, either between linear single-boundary Wiener diffusion processes (Tillman, Van Zandt, & Logan, 2020), or linear independent “ballistic” processes where noise is purely from trial-to-trial variation in evidence and in the distance between the threshold and the starting-point of evidence accumulation (Brown & Heathcote, 2008). Various refinements have been proposed, such as adding trial-to-trial variation to the Wiener diffusion model to address its failure to explain differences between the speed of correct and error RTs (i.e., the popular diffusion decision model; Ratcliff & Rouder 1998), or assuming non-linear and partially dependent evidence accumulation due to neurally inspired leakage and lateral inhibition processes (Usher & McClelland, 2001), albeit at the cost of making parameter estimation more difficult (e.g., Boehm et al., 2018; Miletic, Turner, Forstmann, & van Maanen, 2017).

Logan et al. (2014) proposed that an independent racing-diffusion architecture could be used to model both the go runners (one for each response option) and the stop runner. The single-boundary Wiener diffusion process is often thought of as neurally plausible, and has the further advantage that it produces a Wald distribution (Wald, 1947) of finishing times, which has an analytic likelihood, facilitating Bayesian and maximum-likelihood estimation. In simple and choice RT paradigms, this racing-diffusion model displays good estimation performance (Castro, Strayer, Matzke, & Heathcote, 2019; Tillman et al., 2020). Importantly, as opposed to descriptive models, its parameters—non-decision time ($t_0$), the rate of evidence accumulation (*v*), and the decision threshold (*B*)—are directly informative about the psychological causes of performance differences. For example, Castro et al. (2019) showed that evidence-accumulation rates provide a measure of the limits on attention capacity in the International Organization for Standardization’s “Detection Response Time“ task (ISO, 2015) used to assess how distractions affect driving. Similarly, across a range of evidence-accumulation models it has been found that participants can change the decision threshold to control speed–accuracy trade-offs (e.g., increase the threshold to improve accuracy at the cost of slowing; e.g., Ratcliff & Rouder, 1998), and to take account of prior information (e.g., lowering the threshold corresponding to a more common response; e.g., Garton et al., 2019).

However, Matzke et al. (2020) showed, using Bayesian estimation, that parameter-recovery performance for Logan et al.’s (2014) application of the racing-diffusion model to the stop-signal paradigm is extremely poor. The estimated variability of the stop process tends to zero no matter what its true value is, with associated inconsistencies in the estimates of all other parameters. In particular, in the full model where the starting point of evidence accumulation was assumed to be variable, simulations indicated a severe overestimation of non-decision time and underestimation of the distance between starting point and decision threshold. Matzke et al. also investigated independent racing ballistic evidence processes for all runners where the trial-to-trial variability in the evidence and in the distance from start point to threshold both have lognormal distributions. This lognormal-race model is arguably the simplest and most mathematically tractable evidence-accumulation model (Heathcote & Love, 2012; Rouder, Province, Morey, Gomez, & Heathcote, 2015), but estimation performance, although clearly better than for the racing-diffusion version, was still poor.

For both models, Matzke et al. (2020) attributed these problems to the shift parameter of the stop runner required by the evidence-accumulation models. The timing of the stop runner can only be observed indirectly, through its effect on the (i) probability of successfully inhibiting a response on stop trials; and (ii) difference between the distribution of go RTs on trials without stop signals and the distribution of RTs when inhibition fails on stop trials “signal-respond” RT. In paradigms without a stopping component, the irregularity associated with the shift parameter is typically not problematic, neither when fitting simple descriptive distributions, including the shifted Wald (Heathcote, 2004), nor for the diffusion decision model (Ratcliff & Childers, 2015) and various parametrizations of independent race models (e.g., Castro et al., 2019; Heathcote et al., 2019). The indirect nature of the information about the stop runner means that this is not the case in the stop-signal paradigm.

In the next section, we propose a solution to these estimation problems that maintains the advantages of a psychologically meaningful parametric characterization of the go process and attention failures, while using a descriptive distribution to simultaneously provide good measurement of the key index of inhibitory ability, SSRT. In order to validate this “hybrid” model, we apply it to data from an experiment that combines a stop-signal task and a complex go task design with manipulations that are expected to selectively influence the evidence-accumulation rate and threshold parameters of the go process. We show that a hybrid model parameterized to instantiate these selective influence assumptions provides a relatively simple and accurate account of the data. We then report the results of a parameter-recovery study to demonstrate that even in this complex design, Bayesian estimation of the hybrid model performs well with similar numbers of test trials that are required in standard evidence-accumulation model applications.

**Fig. 2:**
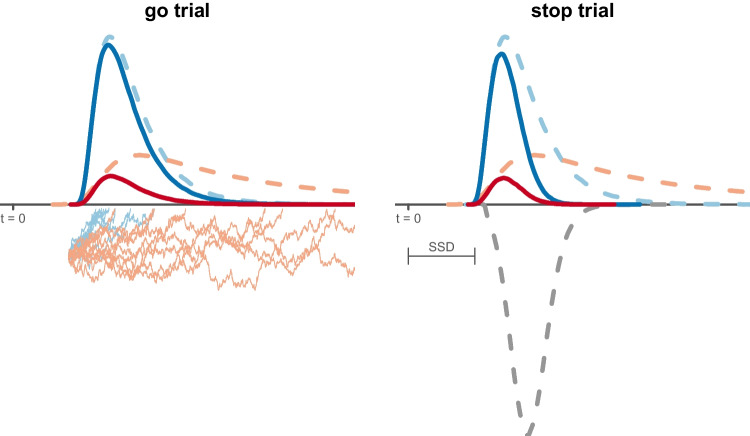
The *left panel* shows the evidence-accumulation process on go trials, with the choice stimulus presented at $t = 0$. Once the stimulus is encoded, two evidence accumulators (runners) corresponding to each response option start to race each other from the same initial level. The runner that crosses its response threshold first wins the race and triggers the associated response. The *jiggly lines* show ten races between the runner that matches (*blue*) and the one that mismatches (*red*) the choice stimulus. Here the thresholds are assumed to be the same and correspond to the time axis, but the thresholds of the two accumulators may differ. The *dashed lines* show the finishing time distribution of each runner across trials, which are longer for the red “mismatch” accumulator as it has a lower average accumulation rate. The *solid lines* show the distribution of observed “winning” times where the corresponding runner finished first; these are shorter than the finishing times because faster runners win races. The *right panel* shows these distributions on stop trials, where the presentation of the choice stimulus is followed at $t =S SD$ by a stop signal that triggers the stop runner. The *dashed lines* show the finishing time distributions, with the *gray line* depicting the ex-Gaussian stop runner. The *solid lines* represent the winning times of the go runners, i.e., the distribution of finishing times where the corresponding go runner was fast enough to beat the other go runner and the stop runner. These winning times are faster for stop than for go trials because slower go finishing times tend to lose the race to the stop runner. The winning times for the stop runner (i.e., finishing times that were fast enough to beat both go runners) are not shown because the stop runner does not produce overt responses, so this distribution is unobserved. SSD: stop-signal delay. Figure available at https://flic.kr/p/2mXYBbh under a CC-BY 2.0 license (https://creativecommons.org/licenses/by/2.0/)

## The hybrid racing-diffusion ex-Gaussian (RDEX) stop-signal model

Figure 2 shows the hybrid racing-diffusion ex-Gaussian (RDEX) stop-signal model. For go trials, it assumes that each of the *N* independent go accumulators is a Wiener diffusion process starting at zero (illustrated by thin irregular lines) with evidence-accumulation rate *v* ($v>0$) and threshold *b* ($b>0$). The finishing time distribution of each accumulator is a Wald distribution (dashed blue and red lines) so that the probability density function (PDF) of the finishing time distribution of go accumulator $i,i= 1,\cdots ,N$, is given by:

1 $$\begin{aligned} f_i(t)= b_i (2\pi t^3)^{-\frac{1}{2}} \exp \left( -\frac{1}{2t} (v_it - b_i)^2 \right) , \end{aligned}$$

for $t>0$. The finishing time distribution of the winner of the race is given by the distribution of the minima of the Wald distributions for all the runners (solid blue and red lines). The model assumes a parameter-dependent lower bound (i.e., non-decision time) for each go accumulator, $t_0$, and a fixed value of 1 for the variability of the diffusion process that makes the model identifiable (Donkin, Brown, & Heathcote, 2009).

To account for fast error RTs, the model can be extended to allow for trial-by-trial variability in start point, where start point is assumed to be uniformly distributed from $0-A$, so that the difference between the threshold and *A* (i.e., threshold gap) is given by $B=b-A$ ($B>=0$) (Logan et al., 2014; Matzke et al., 2020). As errors in the present paradigm were fast and estimating the trial-to-trial variability parameter of the racing-diffusion model is difficult even in standard choice tasks (Castro et al., 2019), here we will assume $A=0$ and hence $B=b$. However, our implementation of the RDEX model (https://osf.io/u3k5f/) in the Dynamic Models of Choice software (Heathcote et al., 2019) allows for *A* to be estimated in case it is useful in other applications. This implementation allows for either one^1^ or two go runners, but it can be easily extended to accommodate more than two response options on the go task by adding extra runners (Logan et al., 2014), and also other evidence-accumulation models, such as the Linear Ballistic Accumulator (Brown & Heathcote, 2008) or the lognormal-race model (Heathcote & Love, 2012; Rouder, Province, Morey, Gomez, & Heathcote, 2015).

On stop trials, the RDEX model assumes that the same evidence-accumulation process assumed for go trials races independently with a stop runner, but it is not committed to any specific accumulation process for the stop component. In particular, the finishing time distribution of the stop runner is described by an ex-Gaussian distribution (dashed grey line in Fig. 2), with PDF:

2 $$\begin{aligned} f(t;\mu ,\sigma ,\tau )= \frac{1}{\tau }\exp \left( \frac{\mu - t}{\tau } + \frac{\sigma ^2}{2\tau ^2}\right) \Phi \left( \frac{t-\mu }{\sigma }-\frac{\sigma }{\tau }\right) , \end{aligned}$$

for $\sigma > 0$, $\tau >0$, where $\Phi$ is the standard normal distribution function, defined as

3 $$\begin{aligned} \Phi \left( \frac{t-\mu }{\sigma }-\frac{\sigma }{\tau }\right) = \frac{1}{\sqrt{2\pi }}\int ^{\frac{t-\mu }{\sigma }-\frac{\sigma }{\tau }}_{-\infty } \exp \left( \frac{-y^2}{2}\right) dy. \end{aligned}$$

To ensure that negative and unreasonably small estimates of the time to complete the stop process are impossible, we use a truncated ex-Gaussian distribution, after re-normalizing the PDF in Eq. 2 as follows:

4 $$\begin{aligned} f(t;l<T<u;\mu ,\sigma ,\tau )= \frac{f(t;\mu ,\sigma ,\tau )}{F(u;\mu ,\sigma ,\tau )-F(l;\mu ,\sigma ,\tau )}, \end{aligned}$$

where

5 $$\begin{aligned} F(t;\mu ,\sigma ,\tau )= \Phi \left( \frac{t-\mu }{\sigma }\right) - \exp \left( \frac{\sigma ^2}{2\tau ^2} - \frac{t-\mu }{\tau }\right) \Phi \left( \frac{t-\mu }{\sigma }-\frac{\sigma }{\tau }\right) . \end{aligned}$$

The mean and variance of the truncated ex-Gaussian distribution can be easily obtained through simulation. In our applications, we assumed an upper bound of $u=\infty$ and a lower bound of $l=.05$ s. The latter setting was motivated by limits on neural transmission times based on Hanes and Carpenter (1999)’s estimate of the onset latency of visual cells in the macaque visuomotor system. However, we found that SSRT estimates were very little effected if we assumed $l=0$.

Although the ex-Gaussian distribution does not directly correspond to an accumulation process, we do not mean to imply by using it that the stop runner is not accumulating evidence about the presence of the stop signal. Rather, it is used as a flexible approximation to such an accumulation process with the advantage that it both facilitates parameter estimation (i.e., makes all parameters in the model identifiable) and interpretation (i.e., it enables estimation of a key quantity of interest, SSRT). Instead of the descriptive ex-Gaussian distribution, we also considered using the Wald distribution with a fixed shift parameter to model the finishing time distribution of the stop runner. Although this approach can improve estimation performance relative to Logan et al.’s (2014) application of the racing-diffusion model, it still requires an impractically large number of trials (see Supplementary Materials in Matzke et al., 2020). A lognormal finishing time distribution for the stop runner with a fixed lower bound performs much better, but the additional benefit in a cognitive-process sense is questionable. In particular, the utility of using a shifted lognormal distribution is in differentiating the decision and non-decision time components of RTs, as it can only differentiate rate and threshold effects on the basis of extra selective influence assumptions. However, decision and non-decision time cannot be differentiated if the shift parameter is fixed. To avoid any temptation to make psychological interpretations of the stop runner’s parameters beyond providing an estimate of the latency of stopping, we prefer to use the ex-Gaussian distribution for describing the stop runner and hence the shape of the SSRT distribution.

Following Matzke et al.’s (2019) mixture likelihood app-roach, the RDEX model can be extended to account for instances when the go or the stop runners were not triggered in response to the go and the stop signals, respectively, which can be interpreted as attention failures. The resulting dynamic multinomial processing tree representation of the RDEX model assumes two additional parameters, $P_{GF}$ quantifying the probability that the go runners were not launched and $P_{TF}$, generically referred to as “trigger failures” (Logan, 1994), quantifying the probability that the stop runner was not launched.

On go trials, the joint likelihood of response *i* at time $T=t$ is given by:

6 $$\begin{aligned}&L_{GO}(\varvec{\theta }_{go_i},\varvec{\theta }_{go_j},P_{GF};t) = P_{GF} + (1-P_{GF}) \nonumber \\&\quad \times f(t;\varvec{\theta }_{go_i}) \times S(t;\varvec{\theta }_{go_j}), \end{aligned}$$

where $f(\varvec{\theta }_{go_i})$ is the PDF of the finishing time distribution of go accumulator *i* with parameters $\varvec{\theta }_{go_i} = (v_i, b_i,t_{0_i})$ and $S(\varvec{\theta }_{go_j})$ is the survival function of the finishing-time distribution of go process *j* with parameters $\varvec{\theta }_{go_j} = (v_j, b_j,t_{0_j})$. $P_{GF}$ is assumed to be independent of SSD and trial type (i.e., go, failed or successful stop trials) and to be the same for the two go runners. On failed stop trials, the joint likelihood of response *i* at time $T=t$ is given by:

7 $$\begin{aligned}&L_{SR}(\varvec{\theta }_{go_i},\varvec{\theta }_{go_j},\varvec{\theta }_{stop}, P_{TF},P_{GF};SSD, t) = \nonumber \\&\qquad (1-P_{GF}) \times \nonumber \\&\qquad \Bigl (P_{TF} \times f(t;\varvec{\theta }_{go_i}) \times S(t;\varvec{\theta }_{go_j}) + \nonumber \\&\qquad (1-P_{TF}) \times f(t;\varvec{\theta }_{go_i}) \times S(t;\varvec{\theta }_{go_j}) \nonumber \\&\qquad \times S(t;\varvec{\theta }_{stop},SSD)\Bigr ), \end{aligned}$$

where $S(t;\varvec{\theta }_{stop})$ is the ex-Gaussian survival function of the finishing time distribution of the stop runner with parameters $\varvec{\theta }_{stop} = (\mu , \sigma ,\tau )$. Lastly, on signal-inhibit trials, the likelihood of a successful inhibition is given by:

8 $$\begin{aligned}&L_S(\varvec{\theta }_{go_i},\varvec{\theta }_{go_j},\varvec{\theta }_{stop}, P_{TF},P_{GF};SSD,t) = \nonumber \\&\qquad P_{GF} + (1-P_{GF})(1-P_{TF}) \times {\int }^\infty _{.05}f(t;\varvec{\theta }_{stop},SSD) \nonumber \\&\qquad \times S(t;\varvec{\theta }_{go_i}) \times S(t;\varvec{\theta }_{go_j})dt, \end{aligned}$$

where $f(t;\varvec{\theta }_{stop})$ is the ex-Gaussian PDF of the finishing time distribution of the stop runner. The lower limit of integration reflects the support of the finishing time distribution of the stop runner, which was set to .05. For details, the reader is referred to Matzke et al. (2019).

The RDEX model is implemented in the Dynamic Models of Choice (DMC) package (Heathcote et al., 2019) and is available at https://osf.io/u3k5f/. DMC provides a step-by-step introduction to response time modeling using Bayesian hierarchical methods using well-documented tutorials that guide users through the process of specifying, fitting, and assessing various response time models, including RDEX. As the code for the RDEX model builds on earlier tutorials, we recommend users, especially those with relatively little modeling experience, to start with the simpler models available in DMC, such as BEESTS, and work their way up to the complex ones, like RDEX.

In the remainder of this paper, we investigate the performance of the RDEX stop-signal model in a paradigm where multiple manipulations were used to selectively influence evidence-accumulation rates and threshold setting in a difficult choice task.

## Application to a complex choice design

Stop-signal task designs are often simple, using easy go tasks without manipulations that affect go responding. Although Matzke et al.’s (2019) extension of BEESTS enables applications to more complex designs with several within-subjects manipulations that affect choice RT and accuracy, the number of parameters which must be estimated quickly becomes unmanageable. This occurs because the ex-Gaussian parameters lack a psychological interpretation, and so there is no a priori basis for constraining their values across conditions. In contrast, the number of parameters that needs to be estimated in complex choice designs can be greatly reduced based on constraints that are either logically entailed by the psychological characterization of choices as being governed by evidence-accumulation processes (Donkin, Brown, & Heathcote, 2011) or are based on assumptions about the selective influence of manipulations on parameters that have been empirically supported in similar choice tasks.

In what follows, we will illustrate the advantages of the RDEX stop-signal model in a complex choice design used in Garton et al. (2019) by adding to it a stop-signal task. First, we show that the model with a priori theoretically motivated constraints provides a good description of empirical data, supporting the validity of the psychological interpretation of the evidence-accumulation parameters in the context of the stop-signal paradigm. We then show, using an extensive parameter-recovery study, that Bayesian estimation of the model is able to provide accurate and precise parameter estimates even in this complex design, confirming the RDEX’s utility as a measurement model.

### Experimental design

**Fig. 3:**
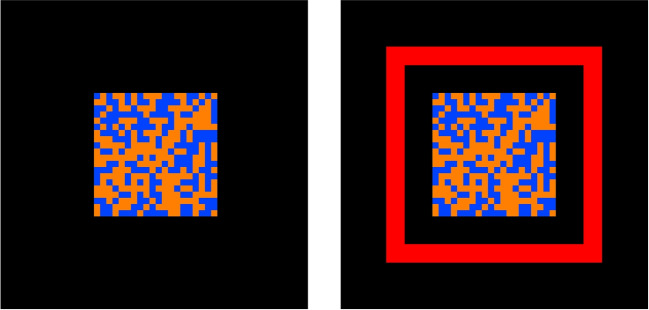
Example of the choice stimulus on go trials (*left*) and the choice stimulus followed by the stop signal on stop trials (*right*). Each cell in the 20 $\times$ 20 checkerboard is randomly colored blue or orange. The difficulty factor determines the proportion of cells colored by the dominant color: 54% in the easy condition and 52% in the hard condition

Garton et al.’s (2019) complex choice design featured a binary choice task with 16 within-subject conditions, corresponding to fully crossing four two-level factors (i.e., a 2 $\times$ 2 $\times$ 2 $\times$ 2 design). The choice was about whether a 20 $\times$ 20 checkerboard stimulus has more blue squares or more orange squares, constituting a *stimulus* factor with levels “blue” vs. “orange“, with the positions of each square varying randomly on each screen refresh. The degree to which one color dominated the image was manipulated over two levels to make up a *difficulty* factor (D: 52% = “hard” vs. 54% = “easy”) that varied unpredictably from trial to trial. The left panel of Fig. 3 shows an example of a choice stimulus on go trials.

Participants were cued on which type of stimulus was more likely on the upcoming trial (70% chance of orange or 70% chance of blue), constituting a *bias* factor (B: “Blue“ vs. “Orange“). The way in which the bias information was presented also varied over two levels, with the same color favored by the cue across a block of trials, or with the favored color varying unpredictably from trial to trial, constituting a *block type* factor (BT: “block” vs. “trial”). For details about the choice task, the reader is refereed to Garton et al. (2019).

Garton et al.’s (2019) complex choice design was extended by adding a stopping component. On a randomly selected 25% of trials, the choice stimulus was followed by a stop signal: a red box appearing around the stimulus array, as shown in the right panel of Fig. 3. The difference between the onset of the choice stimulus and the stop signal, SSD, was set using a staircase algorithm, starting at 0.2 s at the beginning of the experiment, then changing in steps of 0.05 s: SSD increased after successfully inhibited stop trials and decreased after failed inhibitions. See https://youtu.be/kXZk_jCHjkM for a video of the task.

In order to accommodate the variations in speed and accuracy between the 16 conditions in this complex design, BEESTS would potentially have to estimate three ex-Gaussian parameters for each condition and for each of the two choice accumulators, for a total of 3 $\times$ 16 $\times$ 2 = 96 go parameters. Combined with the three ex-Gaussian parameters for the stop runner, and assuming that the parameters for go and trigger failures do not vary over conditions, this requires a total of 101 estimated parameters. In contrast, using the racing-diffusion evidence-accumulation framework for modeling the choice component of the task, we only need 17 go parameters (non-decision time, thresholds, and evidence-accumulation rates), as we explain in detail below. Combined with the three ex-Gaussian parameters for the stop runner, and two parameters for go and trigger failures, the RDEX model requires a total of only 22 parameters, less than a quarter the number of parameters required by the BEESTS model. Perhaps more importantly, the evidence-accumulation parameters support clear psychological interpretations of choice behavior.

### Model parametrization

Table 1 (column Factors) provides an overview of the parametrization in terms of the experimental factors that were assumed to influence each model parameter.

#### Non-decision time

The simplest case is non-decision time, which varies with factors that affect the time to encode the stimulus or to make a response. As none of the manipulations are likely to have these effects, the same non-decision time parameter was assumed for all conditions.

#### Decision threshold

Bias induced by stimulus probability cues is mediated by the threshold of the accumulator corresponding to the more likely response being lowered relative to that for the less likely response. This type of bias is typically assumed to have no effect on evidence-accumulation rates (White & Poldrack, 2014). Allowing for the possibility that the degree of bias favoring blue vs. orange stimuli might differ, four thresholds were estimated, two for the blue and orange accumulators when the bias favors blue, and two when it favors orange. Setting bias is more difficult when prior information differs from trial to trial than maintaining the same setting over a block of trials (Garton et al., 2019). Hence, thresholds were assumed to differ between block types, requiring four estimates for the blockwise and four estimates for the trialwise condition, for a total of eight estimated threshold parameters.

#### Evidence-accumulation rate

As overall accuracy was clearly above chance, the rate for the accumulator that matches the stimulus (e.g., the orange accumulator for majority orange stimuli and the blue accumulator for majority blue stimuli) is necessarily higher than for the accumulator that mismatches the stimuli (i.e., the orange accumulator for blue stimuli and vice versa). Evidence-accumulation rates reflect stimulus characteristics, and so will vary with difficulty. They can also vary as a function of attention, and so can differ between conditions that are more or less attention demanding. Hence, they are likely to differ between block types (Garton et al., 2019). We made the simplifying assumption that evidence-accumulation rates for blue and orange stimuli were the same. As a result, there are also eight evidence-accumulation rates estimates.

#### Stop parameters and go and trigger failures

Performance on cognitive tasks can be impaired due to limits in the quality of the data or limits in available cognitive resources (Norman & Bobrow, 1975). The difficulty manipulation targeted the quality of the data extracted from the go stimulus. In contrast, the stop stimulus was the same between the easy and difficult conditions. We therefore expected that the stop process would be unaffected by the difficulty manipulation, and that the stop parameters would not vary with stimulus difficulty. Although resource limitations might be associated with the greater demands of trialwise than blockwise biasing, stop processing is generally thought to be largely unaffected by the resource demands of the go task (Logan et al., 2014). The go and trigger failure parameters are more likely to be affected by resource demands, but for simplicity we kept both these parameters, just like the stop-runner’s parameters, the same across all conditions. As we show, this did not induce any noticeable misfit.

### Participants and experimental procedure

The sample consisted of 20 University of Tasmania students (70% female, 18–28 years, with mean = 21.4 and SD = 3.2). Participants provided prior written consent to participate and were informed that the research procedures were approved by the UTAS Human Research Ethics Committee (H0015286). The reader is referred to Garton et al. (2019) for further information about the sample^2^.

Participants performed two two-hour sessions: blocks in the first half of each session featured only the complex choice task, with stop signals added for blocks in the second half. Sessions differed in using either blockwise or trialwise cuing, with the order counterbalanced over participants. Here we focus on the stop-signal blocks and do not analyze the go-only blocks, which from the purposes of the present study served only to familiarize participants with the choice task to ensure habitual responding. In each session, the stop-signal blocks started with two 40-trial practice blocks, followed by eight 60-trial experimental blocks, each with 15 stop trials. The trial on which the stop signal appeared was pre-set. Across the two sessions, participants completed 960 trials, with 240 stop trials in total. The response deadline was set to 2 s. On go trials, the choice stimulus was offset as soon as a response was made or the 2 s had passed. On stop trials, the stimulus was displayed until 2 s had passed regardless of the outcome of the trial. Instructions and feedback on go trials emphasized fast but accurate responding to the choice stimulus, and discouraged participants from strategically slowing their responding by waiting for the stop signal to appear. No feedback was provided on stop trials.

We discarded data from two participants who did not perform the task as instructed: one participant slowed across the experiment and had an extreme response rate on stop trials and one participant started very slow but then sped up, producing an atypical RT distribution truncated above by the 2-s response deadline. We removed trials with RTs faster than 0.2 s (.09%).

### Bayesian hierarchical modeling

We used Bayesian hierarchical methods to estimate the posterior distribution of the model parameters. Instead of treating each participant independently, hierarchical modeling allows the parameters of individual participants to be informed by parameters from other participants by modeling their between-subject variability using population-level distributions (e.g., Farrell & Ludwig, 2008; Gelman & Hill, 2007; Lee, 2011; Lee & Wagenmakers, 2013; Matzke et al.,^2013^; Rouder, Lu, Speckman, Sun, & Jiang, 2005). The population-level distribution can be considered as a prior that shrinks the participant-level estimates to the population mean, typically resulting in less variable and, on average, more accurate estimates, especially when the participant-level estimates are relatively uncertain.

**Table 1:** Overview of the model parametrization and the prior setting

Parameter type		Factors	Hyper priors			
			Location	Scale	LB	UB
Go parameters						
$t_0$	Non-decision time	-	.3	5	.1	1
*B*	Decision threshold	BT, B, R	1	5	0	$\infty$
*v*	Evidence-accumulation rate	BT, D, M	2	5	0	$\infty$
Stop parameters						
$\mu$	Mean of Gaussian component	-	.5	1	0	4
$\sigma$	SD of Gaussian component	-	.1	1	0	4
$\tau$	Mean of exponential component	-	.1	1	0	4
Go and trigger failure parameters						
$\Phi ^{-1}(P_{GF})$	Go failure	-	-2	2	-$\infty$	$\infty$
$\Phi ^{-1}(P_{TF})$	Trigger failure	-	-2	2	-$\infty$	$\infty$

*Note.* LB = lower bound. UB = upper bound. BT = block type (blockwise or trialwise). B = Bias (towards Blue or Orange for BLUE and ORANGE accumulators, respectively). R = Accumulator (BLUE or ORANGE). D = Difficulty (easy or hard). M = Accumulator match (true or false, corresponding to the accumulator matching or mismatching the stimulus). The RT data were fit on the seconds scale, so the prior bounds and location and scale parameters also refer to the seconds scale

The participant-level go and trigger failure parameters were projected from the probability scale to the real line with a probit transformation (e.g., Matzke, Dolan, Batchelder, & Wagenmakers, 2015; Rouder, Lu, Morey, Sun, & Speckman, 2008). We assumed (truncated) normal population-level distributions for all parameters, including $\Phi ^{-1}(P_{GF})$ and $\Phi ^{-1}(P_{TF})$, parametrized in terms of their location and scale, with upper (UB) and lower bounds (LB) as shown in Table 1. The population-level location parameters were assigned (truncated) normal hyper priors, with location and scale parameters set to the values shown in Table 1. The population-level scale parameters were assigned exponential prior distributions with a rate of one. These prior distributions are based on previous applications of the original BEESTS model and Logan et al.’s (2014) racing-diffusion stop-signal model. In particular, priors for the stop parameters are similar to the BEESTS priors used by Matzke et al. (2019) for fitting data from a stop-signal experiment that featured a visual stop signal and the manipulation of the difficulty of the go task. Priors for the go parameters were inspired by Matzke et al. (2020) who reported posterior distributions for the racing-diffusion stop-signal model fit to data from Matzke et al. (2019). We refer the reader to the procedure outlined in Heathcote et al. (2019) for using posteriors as a basis for developing priors to be used in the analysis of new stop-signal data.

We used the DMC package (Heathcote et al., 2019) to sample from the joint posterior distribution of the model parameters. Posterior samples were obtained using the differential-evolution Markov chain Monte Carlo algorithm (DE-MCMC; Ter Braak, 2006), which efficiently takes the correlation structure between parameters^3^ into account (Turner, Sederberg, Brown, & Steyvers, 2013). The number of chains was set to three times the number of participant-level model parameters (i.e., 66). The chains were thinned to only keep every 5th sample. Migration was set to occur on a randomly chosen 5% of iterations during the burn-in period, and after this period only cross-over steps were performed. To speed up convergence, start values were based on posterior samples obtained from fitting the data of each participant separately. We assessed convergence by visually inspecting the MCMC chains and computing the Gelman-Rubin convergence statistic ($\hat{R} <1.1$ for all population and participant-level parameters; Brooks & Gelman, 1998; Gelman & Rubin, 1992). After convergence, we obtained an additional 250 samples per chain; these samples were used for inference about the model parameters and the descriptive accuracy of the RDEX presented below. The data and the DMC code for fitting the model and for performing the analyses are available in the Supplementary Materials at https://osf.io/u3k5f/.

The posterior distributions of all group-and participant-level parameters are shown in the Supplementary Materials. The posteriors were all unimodal and well constrained. We used the 95% credible intervals of the participant-level parameters to ascertain that the evidence-accumulation rate and threshold parameters were acting in a way consistent with their psychological interpretations. The credible intervals were computed as the range of values between the $2.5^{th}$ and $97.5^{th}$ percentiles of the posterior distribution of the average of the relevant parameter over participants. We present the results in terms of posterior medians with credible intervals in square brackets.

The difference between evidence-accumulation rates for the accumulators that match and mismatch the stimulus reflect the difficulty of a choice. We found that these differences were clearly larger for easy (2.4 [2.32,2.48]) than hard (1.37 [1.31,1.43]) stimuli (difference = 1.03 [0.93,1.13]). The same pattern held in both the blockwise bias (difference = 0.91 [0.78,1.05]) and trialwise bias (difference = 1.15 [1.01,1.3]) conditions. The difference between these conditions was credible (0.24 [0.04,0.43]), consistent with allowing evidence-accumulation rates to differ between them in the model.

**Fig. 4:**
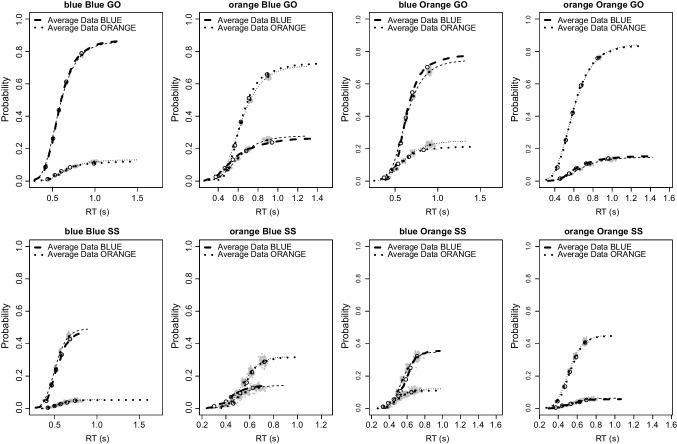
Cumulative distribution functions for the observed go RTs and signal-respond RTs (*thick lines*; *open circles* show the $10^{th}$, $30^{th}$, $50^{th}$, $70^{th}$, and $90^{th}$ percentiles) and for the model fits (*thin lines*; *grey points* show predictions resulting from the 100 posterior predictive samples, with *solid points* showing their average). Each panel contains results for BLUE and ORANGE responses. Panel titles indicate stimulus (blue vs. orange), bias (blue vs. orange), and trial type (GO vs. SS = stop signal). The results are collapsed over the difficulty and block type manipulations

If participants modulate their response bias to take advantage of cues indicating which response is more likely on the upcoming trial, the threshold for accumulators that are incongruent with the bias cue should be higher than for accumulators that are congruent with the bias cue. This was true overall: incongruent = 1.92 [1.88,1.95] vs. congruent = 1.69 [1.61,1.73], with difference = .23 [.21,.24]). The same pattern held in the blockwise bias (difference = .12 [.10,.15]) and trialwise bias (.33 [.30,.35]) conditions. Again, the difference between the two was credible (.20 [.16,.24]), consistent with allowing threshold bias to differ over this factor in the model. These results, together with the excellent goodness-of-fit of the model with theoretically motivated constraints reported below, suggest that estimates of the evidence-accumulation rate and threshold parameters were consistent with their psychological interpretation assumed by the model.

## Assessing goodness-of-fit

We used posterior predictive simulations (Gelman, Meng, & Stern, 1996) to evaluate the descriptive accuracy of the RDEX model. In particular, we compared the observed data to predictions based on the full joint posterior distribution of the participant-level parameter estimates. This procedure simultaneously accounts for sampling error and the uncertainty of the parameter estimates. For each participant, we randomly selected 100 parameter vectors from their joint posterior and generated 100 stop-signal data sets for the current complex choice design using these parameter vectors, the observed SSDs, and the observed number of go and stop trials per cell of the design. We evaluated three features of the data: (1) go RT and signal-respond RT distributions; (2) the probability of responding as a function of SSD (i.e., inhibition function); and (3) the increase in signal-response RTs as a function of SSD.

### Go and signal-respond RT distributions

Figure 4 shows the results of the posterior predictive simulations for the cumulative distribution function (CDF) of go RTs and signal-respond RTs. As the model assumes that going and stopping are independent, signal-respond RTs were collapsed across SSDs.^4^ For brevity, results are collapsed over the difficulty and block type factors. Detailed results for each cell of the design are provided in the Supplementary Materials.

The observed and predicted CDFs were averaged across participants. Note that the upper asymptotes of the CDFs show the probability of a correct and error response; these add to one for go RTs, and to the response rate for signal-respond RTs. Within each panel, thick dashed and dotted lines show CDFs for each observed response (i.e., BLUE vs. ORANGE). The open circles show the 10th, 30th, 50th, 70th, and 90th percentiles of the distributions. Thin dashed and dotted lines show predicted CDFs for each response averaged across the 100 posterior predictive replications, with the black bullets showing the corresponding five percentiles of the distributions. The surrounding clusters of grey dots reflect the uncertainty in the predicted percentiles, showing predictions for each of the 100 posterior predictive samples.

The model generally displays excellent fit and aligns very well with the observed data, especially given the complex design and the relatively parsimonious model parameterization. The only evident misfit is in the CDF of go RTs (top row, third column), where the model slightly under-estimates accuracy for blue stimuli in the Orange bias condition.

### Inhibition and signal-respond RT functions

**Fig. 5:**
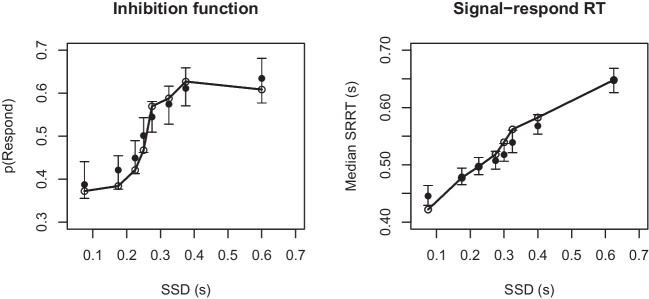
Observed vs. predicted inhibition function (*left panel*) and median signal-respond RTs (*right panel*). Observed data are shown with *open circles joined by lines*. Model fits are shown with *solid points*, with the 95% credible intervals obtained based on predictions resulting from the 100 posterior predictive samples. SSD = stop-signal delay; p(Respond) = probability of responding on stop trials; SRRT = signal-respond RT

The left panel of Fig. 5 shows the results of the posterior predictive simulations for the inhibition function averaged across participants. The right panel of Fig. 5 shows the results of the posterior predictive simulations for median signal-respond RT as a function of SSD, averaged across participants. Note that SSDs are unevenly spaced so that an approximately equal number of data points contributes to each SSD category.

Stop-signal race models predict increasing inhibition and signal-respond RT functions. As expected, the predicted probability of responding increases as SSD increases; stopping becomes more difficult as the interval between the onset of the go and the stop signal increases. The model fits well, with the observed probability of responding falling well within the model’s 95% credible intervals. Although median signal-respond RT is slightly over-predicted ($\approx$ 10ms) by the model at the first SSD, overall the model accurately captures the strong increase with SSD.

These results demonstrate that the RDEX model with a priori theoretically motivated constraints provides a good description of the observed data, even in a complex design, supporting the validity of the evidence-accumulation parameters in the context of the stop-signal paradigm. Had these constraints been inaccurate (i.e., the constrained parameters should have been free to vary, or vice versa), we would expect this to diminish the model’s ability to account for the data and result in model misfit. We now turn to a parameter-recovery study showing that the model provides accurate and precise parameter estimates in our design.

## Parameter-recovery performance

The DMC code used for the parameter-recovery study (Heathcote, Brown, & Wagenmakers, 2015) is available at https://osf.io/u3k5f/. For each of the 18 participants in our study, we generated 200 data sets from the RDEX model using the complex design described above. To ensure that the “true” data-generating values were in a realistic range, we used the posterior means obtained from fitting the model to the data of each participant individually.^5^ The resulting posterior means were used to generate 200 replicate data sets for each participant. Identical to the empirical data, each replicate data set consisted of 960 trials of which 25% were stop trials, and we used the same staircase algorithm with a starting SSD of 0.2 s and step size of 0.05 s.

The RDEX model was then fit to each of the 18 $\times$ 200 data sets to assess how well the true and estimated parameter values correspond. We used the same procedures as before to infer the posterior distribution of the model parameters, with the exception that instead of hierarchical estimation, we fit each data set separately. The start values for each chain were sampled from the prior distributions. As before, we assumed weakly informed (truncated) normal prior distributions for each parameter, with the location and scale parameters, and lower and upper bounds shown in Table 1.

**Fig. 6:**
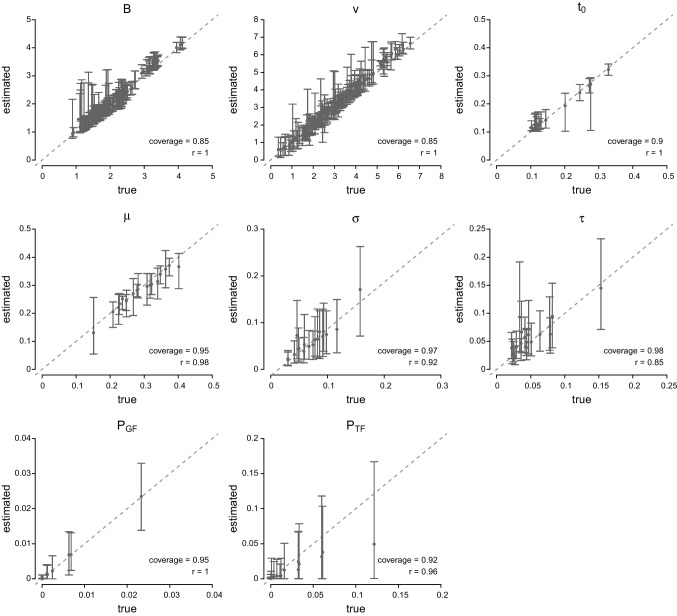
Parameter-recovery results. Results for the go and trigger failure parameters $P_{GF}$ and $P_{TF}$ are presented on the probability scale. See text for details

We summarized recovery performance by computing the correlation between the true and estimated parameter values and the coverage of the posterior distributions across the 200 replications (i.e., the proportion of times the true value falls within the estimated 95% credible intervals). Figure 6 shows scatterplots between the true and the estimated parameter values, with the dashed diagonal showing the identity line. For non-decision time $t_0$ and the stop and the go and trigger failure parameters, each panel shows results for 18 true values, derived from the data of the 18 participants. For *B* and *v*, we combined the eight threshold and eight evidence-accumulation rate parameters in single panels, each showing results for 8$\times$18 true values.

In each panel, the points indicate the average of the posterior means across the 200 replications. The corresponding error bars show the 95% credible interval of the distribution of the posterior mean. Generally, the model recovered the true values very well, with relatively little bias as evidenced by the points falling along the identity line. The trigger failure $P_{TF}$ parameter is slightly underestimated for high values, but the true values fall well within the 95% credible interval of the posterior mean. The coverage values averaged across the 18 parameter sets and the correlation between the true values and the average of the 200 posterior means are shown in the bottom right corners. The correlations between true and estimated values are high, ranging between 0.85 and 1.00. Similarly, the coverage of the posteriors was close to the nominal 95%, ranging between 0.85 and 0.98. These results show that the RDEX approach is well equipped to retrieve the true data-generating parameters, even in the present complex design with relatively sparse data per cell.

### Caveats and solutions

**Fig. 7:**
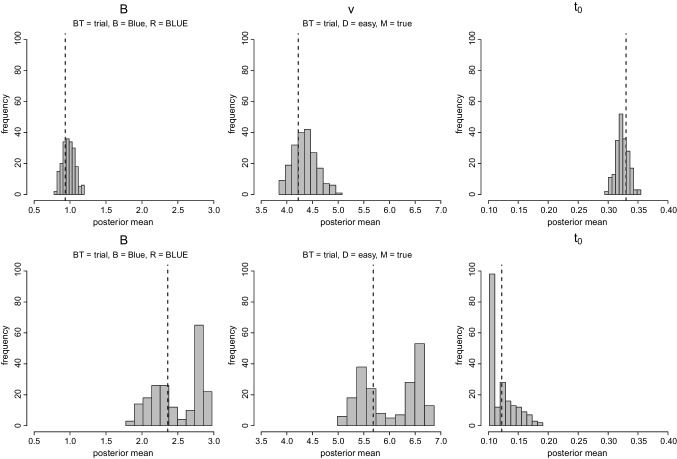
Distribution of the posterior mean across 200 replicated data sets for the threshold *B*, evidence-accumulation rate *v*, and non-decision time $t_0$ parameters. The *vertical dashed lines* show the true values. Block type (BT) = trialwise, bias (B) = blue, accumulator (R) = BLUE, difficulty (D) = easy, and accumulator match (M) = true, corresponding to the accumulator matching the stimulus. The *top row* shows a parameter setting resulting in excellent parameter recovery, where the distribution of posterior means is unimodal and centered around the true data-generating value for all parameters. The *bottom row* shows a parameter setting with poor recovery, with bimodality in the distribution of the posterior means of the go parameters. For both parameter settings, the figure shows three out of the 22 parameters, one for each parameter type (i.e., evidence-accumulation rate, threshold, and non-decision time). The full parameter set for all 18 parameter settings are presented in the Supplementary Materials

Figure 6 shows that the recovery performance of the RDEX model was, in general, excellent. However, as illustrated in the Supplementary Materials, in certain parameter regions, the sampling distribution of the posterior mean of the go parameters showed some irregularities. In particular, for each true parameter setting, we plotted the distribution of the posterior means across the 200 replications. In the majority of parameter settings, the distribution of the estimated posterior means was unimodal and centered around the true value, indicating good recovery. This is illustrated in the top panel of Fig. 7, showing the sampling distribution of the posterior mean for *B*, *v*, and $t_0$ for a representative data-generating parameter set. This particular setting was chosen because it featured parameters that were average relative to the range of data-generating values we explored. However, as shown in the bottom panel of Fig. 7, for two parameter settings, the sampling distributions of *B*, *v*, and $t_0$ were bimodal, with one peak at the true values, and the estimated posterior means for $t_0$ stacking up against the lower bound of the prior (i.e., .1).^6^ For another setting, there was bimodality in *v* and *B*, and for one setting only in *v*, again with one mode being around the true value.^7^ This undesirable behavior was not accompanied by convergence problems. These occasional irregularities only appeared in the go parameters; the sampling distributions of the stop, and go and trigger failure parameters were unimodal and well behaved in all parameter settings. These problems were not resolved by using non-standard settings of the DE-MCMC sampler’s tuning parameters (e.g., enabling it to better explore multi-modal parameter distributions, as suggested by Ter Braak, 2006).

**Fig. 8:**
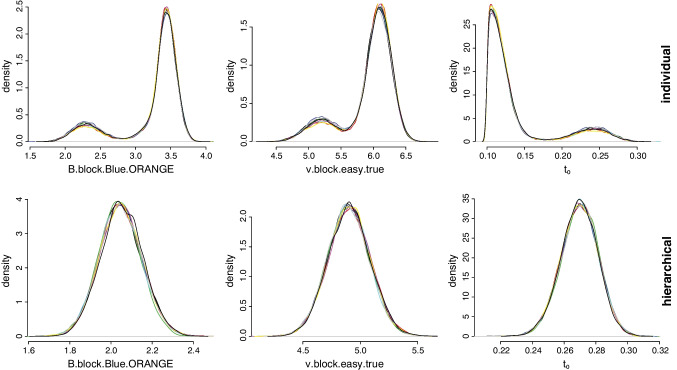
Posterior distributions for nine replications (shown with different colors) of fitting the RDEX model to empirical data. The *top row* shows the posterior distributions resulting from individual estimation, and the *bottom row *shows the posterior distributions of the same participant resulting from hierarchical estimation. For brevity, only three of the 22 parameters are presented: *B*, *v*, and $t_0$. Block type = blockwise, bias = blue, accumulator = orange, difficulty = easy, and accumulator match = true, corresponding to the accumulator matching the stimulus

We also examined this behavior in the empirical data. In particular, we fit the data of each participant nine times, using different start values and both individual as well as hierarchical estimation, to evaluate the consistency of the results across different sampling runs. As shown in the Supplementary Materials, the posterior distributions resulting from individual fitting showed consistent results, with the nine sets of posteriors being virtually indistinguishable from one another. For four participants, however, all nine sampling runs resulted in bimodal $t_0$, *B* and *v* posterior distributions, similar to the sampling distributions observed in the parameter-recovery study. The top panel of Fig. 8 shows the posterior distributions of one of the problematic participants resulting from individual estimation. Note that as opposed to the simulations, here we see the bimodality directly in the posterior distributions, likely reflecting the decreased resolution of the posteriors resulting from noise in the real data. The fact that the bimodality is present in the posterior is, of course, desirable because even a single sampling run can alert researchers to potential spurious estimates.

Even more desirable is the result that hierarchical estimation, where constraints are provided by the group level, circumvents this issue altogether. As shown in the Supplementary Materials, the posterior distributions resulting from hierarchical fitting not only showed consistent results across the nine sampling runs, but it also resulted in well-behaved unimodal posteriors for all participants. The bottom panel of Fig. 8 shows the posterior distributions of the same participant resulting from hierarchical as opposed to individual estimation. The nine posterior distributions are all unimodal. In the case of $t_0$, this mode is away from the lower prior bound and is clearly more plausible. These results indicate that even in problematic parameter regions, hierarchical estimation of the RDEX model results in consistent and well-behaved parameter estimates.

## Discussion

Cognitive psychometrics seeks to characterize behavior in terms of parameters that correspond to latent psychological constructs. For example, decision thresholds correspond to response caution. Those processes have well-defined and psychological interpretations that follow from the processes described by the model. For example, more cautious responding arises from requiring more evidence before triggering a response by setting a higher average threshold over accumulators. When successful, the benefits of this approach are twofold (Heathcote, 2019).

First, the range of potential model parameterizations is simplified, both from a priori constraints related to the psychological interpretation of the parameters and from previous findings in related paradigms that can reasonably be assumed to engage the same psychological mechanisms. As an example of the former type of constraint used in the present experiment, thresholds in an evidence-accumulation model are set strategically and cannot vary with factors whose levels change unpredictably. As such, threshold differences cannot explain the effects of the manipulation of the difficulty of the choice stimuli, because difficulty varied unpredictably from trial to trial. Instead, choice difficulty must be explained by differences in evidence-accumulation rates as they are determined, at least in part, by stimulus-related factors. As an example of the latter type of constraint, we accounted for response bias with threshold changes consistent with findings such as those of White and Poldrack (2014) and Garton et al. (2019). The validity of the model (i.e., the assertion that its parameters measure the psychological processes to which the model claims they correspond) can be determined by its ability to successfully fit data when restricted to such simple parameterizations. In the present case, the validity of the RDEX model was supported on these grounds.

A second advantage is that estimated parameter values can be used to further test the model’s validity and to draw clear inferences about psychological processes. As an example of the former, the RDEX model was validated in the application reported here because, as expected, threshold parameters were lower for the accumulator favored by the bias cues. Similarly, the difference in evidence-accumulation rates between the accumulator that matches the stimulus and the one that mismatches the stimulus—which indexes the degree of discrimination between stimuli—was larger for easier than harder stimuli. As an example of the latter, Heathcote et al. (2022) compared the performance of the younger participants reported here to the performance of older participants performing the same task. Their analysis identified multiple mechanisms causing the pervasive phenomenon of age-related slowing (Salthouse, 1996), with older participants having increased non-decision time, more cautious responding as indexed by thresholds averaged over accumulators, and slower processing speed as indexed by the average of matching and mismatching evidence-accumulation rates. It also enables the relative influence of each mechanism on different aspects of behavior to be determined (see Strickland, Loft, Remington, & Heathcote, 2018), in this case showing that non-decision time and processing speed played major roles and caution a more minor role.

However, garnering these benefits requires overcoming trade-offs that can threaten parameter interpretability. A primary threat comes from the strong correlations between parameters that often occur in cognitive models, particularly in more complex designs. Although the simplified parameterizations intrinsically afforded by cognitive models help in this regard, further constraint is often needed. Two such sources of constraint are increasingly relied upon in cognitive psychometrics. The first, hierarchical Bayesian estimation, leverages the assumption that participants behave in similar ways to create informative priors that reduce the variance of individual parameter estimates. The second solution requires the cognitive psychometric model to account for dynamic aspects of choice behavior such as RT. Such accounts often assume a race among processes corresponding to each choice (Logan & Cowan, 1984), with the runners modeled by evidence-accumulation processes (Heathcote et al., 2019). Here we addressed a challenge to the latter solution posed by the need to estimate parameter-dependent lower bounds (or “shifts”) required by evidence-accumulation models. We did so in the context of a particularly difficult case, the stop-signal paradigm. This case is difficult because responses are to be withheld when the runner associated with a stop signal wins the race, and so no RT is observed. Matzke et al. (2020) showed that, as a result, models in which the stop runner is an evidence-accumulation process are not mathematically identified and have poor psychometric properties.

We addressed the lower-bound problem in the stop-signal paradigm with a “hybrid” architecture, combining a descriptive account of the stop runner with an evidence-accumulation account of the go runners representing the choice element in the task. The lower-bound problem is avoided because the descriptive ex-Gaussian stop runner does not require the estimation of a shift parameter. At the same time, retaining evidence-accumulation processes for the go component garners the benefits of a cognitive-process account, such as a simplified parameterization, tests of the model’s validity, and the unambiguous psychological interpretation of the parameter estimates. Importantly, the descriptive ex-Gaussian account of the stop runner still provides an estimate of the distribution of the key index of inhibitory performance in the stop-signal paradigm, stop-signal RT.

Although the hybrid architecture avoided the problems reported by Matzke et al. (2020), we encountered a new estimation problem in certain parameter regions. In particular, individual estimation sometimes resulted in bimodal posterior distributions (in real data) and bimodal sampling distributions of the posterior mean (in simulations) of the evidence-accumulation parameters. Fortunately, the extra constraint provided by hierarchical estimation addressed this problem. Hence, even in these challenging cases, the combination of Bayesian estimation with a generally well-identified cognitive model provides a viable way of bringing the benefits of dynamic cognitive psychometrics to a complex stop-signal design.

Although our simulations showed that the Bayesian hierarchical implementation of the RDEX model requires only a modest number of test trials for accurate estimation, we acknowledge that parameter recovery is a complex issue. It does not only depend on the number of trials and participants, but also on the inferential goals (group vs. individual-level inference, and group comparison vs. individual differences), the parameter region, as well as the experimental design and the corresponding parameterization. We urge readers to use the parameter-recovery module included in the DMC package to conduct their own simulations tailored to the particular situation at hand. Similar considerations are at play for model-recovery performance. The question whether the data-generating model can be recovered from among a set of candidate models is additionally also influenced by the predictions made by the competing models and the model selection measure one wishes to use. Luckily, DMC allows users to conduct their own model-recovery studies using various model selection measures, including the DIC (Spiegelhalter, Best, Carlin, & van der Linde, 2002) and Bayes factors computed via Warp-III bridge sampling (Gronau, Heathcote, & Matzke, 2020).

The tension between process realism and practically useful measurement properties is not unique to the effects of an estimated lower-bound on likelihood-based estimation addressed here. For example, independent race models have been criticized as lacking neural realism, giving rise to interactive racing evidence-accumulation models for both the stop-signal paradigm (Boucher, Palmeri, Logan, & Schall, 2007; Logan et al., 2015) and choice RT paradigms more broadly (Usher & McClelland, 2001). For interactive stop-signal models, single-cell recordings are required to constrain estimation, so their purview in a psychometric sense is generally limited to non-human primates. Fortunately, the independent model has been shown to provide an excellent approximation with respect to behavioral data alone, supporting the validity of many applications (Verbruggen et al., 2019). With respect to choice RT, the independence assumption has also been criticized as being unable to account for the behavioral effects of similarity among choices (Teodorescu & Usher, 2013). Hence, where these effects are present, they potentially compromise psychometric applications of independent race models. However, estimation of the interactive model based on behavioral choice RT data is problematic (Miletic et al., 2017). Fortunately, as in the present case, this problem has proved amenable to a model-based solution, with an elaborated independent-race architecture having been shown to accommodate the similarity effects while maintaining good estimation properties (van Ravenzwaaij, Brown, Marley, & Heathcote, 2019; Miletić et al., 2021).

In summary, all scientific models necessarily make simplifications and use approximations in order to be useful (Box, 1976). The tension between process realism and practically useful measurement properties particularly besets cognitive psychometrics, because of its emphasis on using parameter estimates to quantify latent psychological processes. Our results show that hybrid cognitive architectures like the one proposed here provide a useful new direction in enabling the broader application of cognitive psychometrics, especially in the domain of dynamic response time data.

## Data Availability

The empirical data, the software implementation of the RDEX model, including the code for fitting the data and replicating the parameter-recovery studies are available at https://osf.io/u3k5f/.

## Footnotes

1 Go tasks requiring a non-choice response (e.g., stimulus-onset detection) is typically discouraged due to the likelihood of anticipatory responses (Matzke et al., 2019; Verbruggen et al., 2019), but a model with a single go runner can be appropriate when the choice task is so easy that there are very few errors. In this case, error responses are discarded and the remaining correct RTs are modeled with a single go runner (e.g., Matzke et al., 2013).

2 Garton et al. (2019) also recruited 20 older participants; the data of these participants are not presented in this paper.

3 The correlations among parameters reflect the mathematical structure of the model and are themselves not problematic. However, parameter correlations make estimation more difficult because older MCMC sampling methods tend to be inefficient in the presence of strongly correlated parameters.

4 Note also that early and late SSDs produced by the staircase algorithm typically result in scarce data, which makes collapsing the distributions across SSDs the only practically viable option for assessing descriptive accuracy.

5 To ensure that the recovery results generalize to other parameters settings and not only to those obtained from real data (Matzke et al., 2020), we explored various other true values obtained from perturbing the estimated posterior means. The results were qualitatively similar to the ones presented here.

6 We obtained similar results with lower and higher bounds.

7 The problem with only *v* most likely results from low error rates or the quality of information captured in error responses in that particular parameter region as it only appeared for the *v* parameter associated with the runner that mismatched the stimulus. In the other cases, we suspect that the problems arose as a result of irregularities associated with likelihood-based estimation in the presence of parameter-dependent lower bounds (i.e., $t_0$). Although, as we explained earlier, irregularity is typically not problematic in tasks with observed responses (such as the go task), it can sometimes lead to inconsistent estimates.
